# Acute Effects of High-Intensity Aerobic Exercise on Motor Cortical Excitability and Inhibition in Sedentary Adults

**DOI:** 10.3389/fpsyg.2022.814633

**Published:** 2022-03-17

**Authors:** Ashlee M. Hendy, Justin W. Andrushko, Paul A. Della Gatta, Wei-Peng Teo

**Affiliations:** ^1^Faculty of Health, School of Exercise and Nutrition Sciences, Institute for Physical Activity and Nutrition (IPAN), Deakin University, Geelong, VIC, Australia; ^2^Brain Behaviour Laboratory, Department of Physical Therapy, Faculty of Medicine, The University of British Columbia, Vancouver, BC, Canada; ^3^Motor Behaviour Laboratory, Physical Education and Sports Science Academic Group, National Institute of Education, Nanyang Technological University, Singapore, Singapore

**Keywords:** high-intensity interval training (HIIT), brain-derived neurotrophic factor (BDNF), transcranial magnetic stimulation (TMS), neuroplasticity, extensor carpi radialis

## Abstract

Transcranial magnetic stimulation studies have demonstrated increased cortical facilitation and reduced inhibition following aerobic exercise, even when examining motor regions separate to the exercised muscle group. These changes in brain physiology following exercise may create favorable conditions for adaptive plasticity and motor learning. One candidate mechanism behind these benefits is the increase in brain-derived neurotropic factor (BDNF) observed following exercise, which can be quantified from a venous blood draw. The aim of this study was to investigate changes in motor cortex excitability and inhibition of the upper limb, and circulating BDNF, following high-intensity interval training (HIIT) on a stationary bicycle. Nineteen sedentary adults participated in a randomized crossover design study involving a single bout of high-intensity interval cycling for 20 min or seated rest. Venous blood samples were collected, and transcranial magnetic stimulation (TMS) was used to stimulate the extensor carpi radialis (ECR), where motor evoked potentials (MEP) were recorded pre- and post-condition. Following exercise, there was a significant increase (29.1%, *p* < 0.001) in corticospinal excitability measured at 120% of resting motor threshold (RMT) and a reduction in short-interval cortical inhibition (SICI quantified as 86.2% increase in the SICI ratio, *p* = 0.002). There was a non-significant (*p* = 0.125) 23.6% increase in BDNF levels. Collectively, these results reflect a net reduction in gamma aminobutyric acid (GABA)ergic synaptic transmission and increased glutamatergic facilitation, resulting in increased corticospinal excitability. This study supports the notion that acute high-intensity exercise provides a potent stimulus for inducing cortical neuroplasticity, which may support enhanced motor learning.

## Introduction

Exercise offers many health benefits ranging from extending the span of healthy living to reducing and delaying the onset of several chronic conditions and diseases (Ruegsegger and Booth, 2018). Transcranial magnetic stimulation (TMS) studies have also demonstrated the effectiveness of exercise for increasing cortical facilitation and reducing cortical inhibition to non-exercised limbs, both of which are known precursors to motor training-related neuroplasticity (Kida and Mitsushima, 2018).

Brain-derived neurotropic factor (BDNF) is a molecule that plays a large role in neuroplasticity due to its ability to promote synaptic transmission between neighboring neurons (Miranda et al., 2019). BDNF plays a vital role in regulating synapses, where enhancing BDNF concentrations increases the formation of dendritic spines, which facilitate new synaptic formations between neurons (Lu et al., 2014; Yang et al., 2020). In addition to BDNF, other important neurotrophic factors for promoting neuroplasticity include nerve growth factor (NGF), which plays a role in the survival, growth, and differentiation of peripheral and central neurons (Hall et al., 2018), glial cell line-derived neurotrophic factor (GDNF), also known for its role in neuronal survival in the central and peripheral nervous system and promotes recovery of damaged axons at the neuromuscular junction (Cortés et al., 2017). Vascular endothelial growth factor (VEGF) promotes the growth of blood vasculature and can therefore increase neurovascular coupling (Kou et al., 2019) and finally, insulin-like growth factor 1 (IGF-1) promotes anabolic growth in nearly all bodily tissues (Wrigley et al., 2017). BDNF in particular is known to acutely enhance glutamatergic and reduce gamma aminobutyric acid (GABA)ergic synaptic transmission, thereby altering the excitation/inhibition balance in the brain by increasing the concentration of glutamate and reducing GABA concentrations (Gottmann et al., 2009; Park and Poo, 2013).

Given that a negative relationship exists between levels of BDNF and GABA in the brain (Müller et al., 2020) and that previous literature has demonstrated an acute reduction in GABA correlates with improved motor learning (Stagg et al., 2011), it is conceivable that exercise may be an effective method to “prime” the nervous system by increasing levels of BDNF, which cause a cascading effect on important precursors to promote motor training-related neuroplasticity.

Previous research has shown that exercise can enhance systemic BDNF levels in older adults (Inoue et al., 2020), in resistance trained (Church et al., 2016), and sedentary individuals (Nofuji et al., 2012). This effect has been documented in studies that have investigated both high-intensity (Marquez et al., 2015; Marinus et al., 2019) and moderate intensity aerobic exercise (Inoue et al., 2020), in addition to resistance training (Church et al., 2016). High-intensity aerobic exercise is also known to facilitate motor skill acquisition (Mang et al., 2014; Statton et al., 2015) and retention (Roig et al., 2012), which may be due to the acute increase in systemic levels of BDNF. However, post-exercise BDNF levels are known to differ between physically active and sedentary individuals (Nofuji et al., 2012), where those that are physically active have greater systemic increases, which may explain differences in motor cortex plasticity with motor learning between cohorts (Cirillo et al., 2009). Additionally, physical activity levels have also been shown to alter the impact of exercise on TMS measures of facilitation and inhibition (Lulic et al., 2017). One study showed an acute bout of high-intensity cycling resulted in increased excitability, reduced inhibition, and improved motor performance in a ballistic thumb task; however, the activity levels of participants were not reported (Opie and Semmler, 2019). No increase in corticospinal excitability was reported following either high or moderate intensity exercise in low-fit individuals (El-Sayes et al., 2020); however, inhibitory measures were significantly reduced following both high (Stavrinos and Coxon, 2017) and moderate intensity cycling protocols (Singh et al., 2014; Smith et al., 2014), where participants were either sedentary or of unknown fitness status.

There are currently no studies that have investigated the impact of high-intensity exercise on both BDNF concentrations and TMS measures of cortical excitability and inhibition in sedentary adults. Therefore, the purpose of this study was to investigate changes in the concentration of blood BDNF, motor cortex excitability, and inhibition of the upper limb following high-intensity aerobic interval training performed on a stationary bicycle in sedentary adults, with the goal of gaining valuable insight into how exercise may be utilized in future research for enhancing motor learning and/or rehabilitation. It was hypothesized that following high-intensity interval training (HIIT), intracortical inhibition would be reduced, concomitant with an increase in circulating levels of BDNF. In addition, exploratory analyses were also carried out on other important neurotrophic molecules for promoting preferential neuroplasticity including NGF, GDNF, VEGF, and IGF-1.

## Materials and Methods

### Participants

A total of 19 healthy participants (age 22.6 ± 3.0 years, height 170.2 ± 10.5 cm, and weight 67.4 ± 19.1 kg, 10 female) took part in the study, which was a sub-component of a larger project investigating combined exercise and transcranial direct current stimulation (tDCS). Data presented here are from control conditions where tDCS was not applied. The study was conducted according to the Declaration of Helsinki. Informed consent was obtained from all participants prior to completing the study. To be eligible for inclusion, participants were sedentary, as defined by participating in <150 min physical activity per week for the 6 month period preceding participation. The Exercise and Sports Science Australia (ESSA) adult pre-exercise screening system was conducted, with participants required to score < 2 (low risk of exercise-induced cardiovascular complication) in order to be eligible participate in HIIT. The adult TMS safety screening questionnaire was administered, participants with contraindications to brain stimulation were excluded.

### Study Design

Participants attended the laboratory on two occasions separated by a minimum 1-week washout period, in a randomized crossover design. Due to the nature of the exercise intervention, double blinding was not possible; however, single blinding of the researcher delivering TMS and all analysis procedures was utilized to reduce the potential for bias. All sessions were conducted at the same time of day (same start time), based on the participants preference and availability. All outcome measures were assessed PRE (prior to beginning exercise) and POST (20 min following the cessation of HIIT). The duration of a single session did not exceed 2 h.

### Exercise Intervention

Exercise involved 20 min of HIIT on a stationary bicycle (Marquez et al., 2015). Participants were fitted with a heart rate (HR) monitor, and began with the first 2 min interval (warm up) by cycling at self-selected cadence, while the researcher manipulated the ergometer resistance to achieve a HR of 120 beats per min (bpm). The target HR was then set to 80% of the participant’s estimated maximum (220-age) to begin the first 2 min work period. Cycling speed was increased to 100 revolutions per minute (rpm), and the researcher increased the resistance to achieve the desired HR. A total of 5 × 2 min work periods were completed, each separated by 2 min active recovery, where the participant returned to self-selected cadence at zero resistance. The participant’s HR was monitored throughout, and recorded at the end of every minute (work and rest), and the participants self-rating of perceived exertion (RPE) was recorded at the end of each 2 min period using Borg’s 6–20 scale (Borg, 1998).

### Surface Electromyography and Transcranial Magnetic Stimulation

Surface electromyography (sEMG) of the right extensor carpi radialis (ECR) was recorded using Ag-AgCl electrodes with an inter-electrode distance of 20 mm. The data were recorded in LabChart 8 through a Powerlab 8/30 laboratory analog-digital interface (ADinstrument, Australia). Bandpass filtering was applied (13–1,000 Hz), and sampling occurred at 2,000 Hz. Prior to TMS delivery, the maximal compound wave (Mmax) was elicited through stimulation of the radial nerve, using a DS7AH constant current stimulator (Digitimer, Hertforshire, United Kingdom).

All TMS was delivered with a 70 mm figure-eight coil and a BiStim2 system (Magstim, United Kingdom). The coil was positioned tangential to the scalp with the handle pointing backward at 45° lateral to the midline. To ensure consistent placement of the coil, a cap marked with a 1 cm matrix was fitted in reference to the nasion-inion and inter-aural lines. The optimal site for stimulation of the right ECR was determined through exploration, marked on the cap, and recorded for the subsequent session. Resting motor threshold (RMT) was obtained when a minimum of 6 out of 10 motor evoked potentials (MEP) reached a peak-to-peak amplitude of >50 μV (Rossini et al., 2015). Corticospinal excitability was assessed by delivering 15 single pulse stimuli at intensities of 120% RMT (RMT_120_) and 140% RMT (RMT_140_) and expressed as a percentage of the maximal compound wave. A paired-pulse paradigm was used to assess SICI, involving a sub-threshold conditioning stimulus (80% RMT) and a supra-threshold test pulse (120% RMT) delivered with an interstimulus interval (ISI) of 3 ms. The mean amplitude of the resulting 15 paired-pulse MEPs (MEP_SICI_) was then expressed as a percentage of the mean MEP_120_ using the following equation:


$$SICI_{\left(\%\right)}=\frac{MEP_{SICI}}{MEP_{120}}\times 100$$

Importantly, an increase in the SICI_(%)_ indicates a reduction in intracortical inhibition.

### Blood Samples and Analysis

Venous whole blood samples were drawn from the median cubital vein in a subgroup of 11 participants (age 22.4 ± 3.5 years, six female), prior to the intervention and at 20 min post-exercise (or following 20 min rest). The samples were collected in a 4 ml EDTA vacutainer (BD, North Ryde, NSW) and immediately centrifuged at 12,000 *g* for 10 min at 4°C. Plasma was then frozen at −80°C for later analysis. The multiplex immunoassay was performed by Crux Biolab (Melbourne, Australia). The plasma samples were assayed with a custom-designed Quantibody human-specific protein array (RayBiotech, United States) to detect analyses (BDNF, b-NGF, GDNF, IGF-I, and VEGF) according to the manufacturer’s protocol.

The fluorescently labeled array was analyzed using a GenePix 4000b (Molecular Devices) scanner. Four replicate values were extracted using the GenePix Pro Version 4.0 software, and any outlier was removed and a mean value for each target was provided. The data underwent an intra- and inter-slide normalization process analyzed using the RayBiotech Custom Q-Analyzer software to compare all data across all the arrays. From this data, standard curves were drawn and the raw data were interpolated to generate the amount of each target in the concentration in pictograms per milliliter of each protein in the samples.

### Statistical Analysis

All data were screened for outliers by examination of studentized residuals for values greater than ±3, and Shapiro–Wilk’s test of normality was applied to determine normal distribution of all variables (all *p* > 0.05). Three-way repeated measures ANOVA was used to determine the effect of within-subject factors condition (control and HIIT), time (PRE and POST), and stimulation (RMT_120_ and RMT_140_) on corticospinal excitability, while a two-way ANOVA (condition × time) was used for all other dependent variables. Data are mean ± SD, unless otherwise stated. All statistical analyses were conducted in SPSS 24 (IBM, United States), with significance set at *p* < 0.05. Effect sizes were interpreted using the partial eta squared (*ɳ*_p_^2^) as small (>0.02), medium (>0.13), or large (>0.26; Bakeman, 2005).

## Results

### Heart Rate and RPE

No adverse effects were reported during HIIT exercise. The participants HR and RPE during the HIIT intervention are presented in Table 1. Based on the mean participant age, target HR during work periods was 155 bpm. Responses to the Borg RPE scale during 2 min work bouts ranged from 12 (*somewhat hard*) to 19 (*extremely hard*). Mean RPE during the first rest period was 8.6 ± 1.7, and during the last rest period was 10.3 ± 1.6.

**Table 1:** Heart rate (HR) and rating of perceived exertion (RPE) during work periods of the high-intensity interval training (HIIT) cycling bout; bpm, beats per minute.

	HIIT	
	HR (bpm)	RPE
3–4 min	141 ± 13.5	12.8 ± 1.7
7–8 min	148.3 ± 12.9	13.8 ± 1.8
11–12 min	152.5 ± 11.0	14.3 ± 1.4
15–16 min	153.1 ± 9.1	14.6 ± 2.0
19–20 min	156.1 ± 9.1	15.1 ± 1.7

### Transcranial Magnetic Stimulation

No adverse reactions to TMS were reported. There was no significant condition × time interaction for stimulator output [*F*_(2,15)_ = 0.035, *p* = 0.855, *ɳ*_p_^2^ = 0.002]. The mean stimulator output (%) required to evoke RMT was 34.1 ± 6.2 for the control condition and 34.8 ± 6.8 for the HIIT condition. There was also no significant condition × time interaction for M_max_ [*F*_(2,15)_ = 0.272, *p* = 0.610, *ɳ*^2^ = 0.018]. The peak-to-peak amplitude of the Mmax during the control condition was 8.4 ± 2.8 mV (pre) and 8.4 ± 2.8 mV (post), and 8.0 ± 3.3 mV (pre) and 7.8 ± 3.1 mV (post) for the HIIT condition.

**Figure 1:**
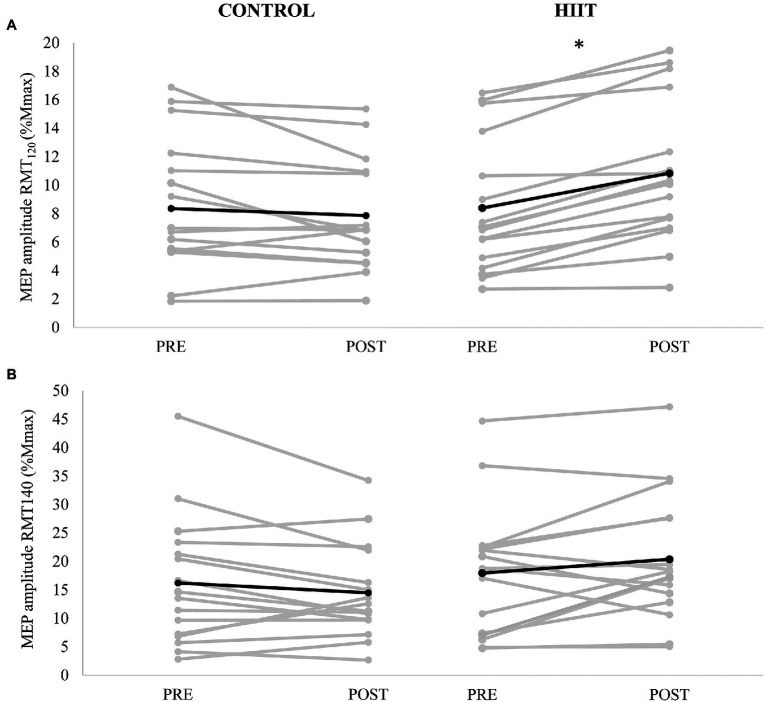
Motor evoked potential (MEP) amplitude (mV) evoked at **(A)** 120% resting motor threshold (RMT) and **(B)** 140% RMT measured PRE and POST for each participant (gray lines) with control condition shown in the left panels, and HIIT shown on the right panels. The mean amplitude for each condition is depicted by the black line. * denotes a significant condition × time interaction (*p* < 0.001).

Cortical excitability, as measured by MEP_120_ and MEP_140_ is shown in Figures 1A,B respectively. There was no significant three-way condition × time × stimulation interaction [*F*_(2,15)_ = 0.228, *p* = 0.640, *ɳ*_p_^2^ = 0.015]. There was a significant two-way condition × time interaction [*F*_(2,15)_ = 11.625, *p* = 0.004, *ɳ*_p_^2^ = 0.437] but no significant interaction for condition × stimulation [*F*_(2,15)_ = 0.009, *p* = 0.927, *ɳ*_p_^2^ = 0.001] or time × stimulation [*F*_(2,15)_ = 0.695, *p* = 0.418, *ɳ*_p_^2^ = 0.044]. A significant simple main effect for stimulation was found [*F*_(2,15)_ = 28.774, *p* < 0.001, *ɳ*_p_^2^ = 0.657]; thus, data were collapsed for stimulation (RMT_120_ and RMT_140_) and a Bonferroni correction applied for multiple comparisons (alpha set at *p* < 0.025). For MEP_120_, a significant condition × time interaction for MEP_120_ [*F*_(2,15)_ = 15.783, *p* < 0.001, *ɳ*_p_^2^ = 0.513]. For the control condition, there was a 6.0% reduction in MEP amplitude (from 8.3 ± 4.8% Mmax to 7.9 ± 3.8% Mmax). For the HIIT condition, there was a 29.1% increase in MEP amplitude (from 8.1 ± 4.5% Mmax to 10.4 ± 4.9% Mmax). There was no significant condition × time interaction for MEP_140_ [*F*_(2,15)_ = 4.300, *p* = 0.056]; a medium effect size was noted (*ɳ*_p_^2^ = 0.223).

**Figure 2:**
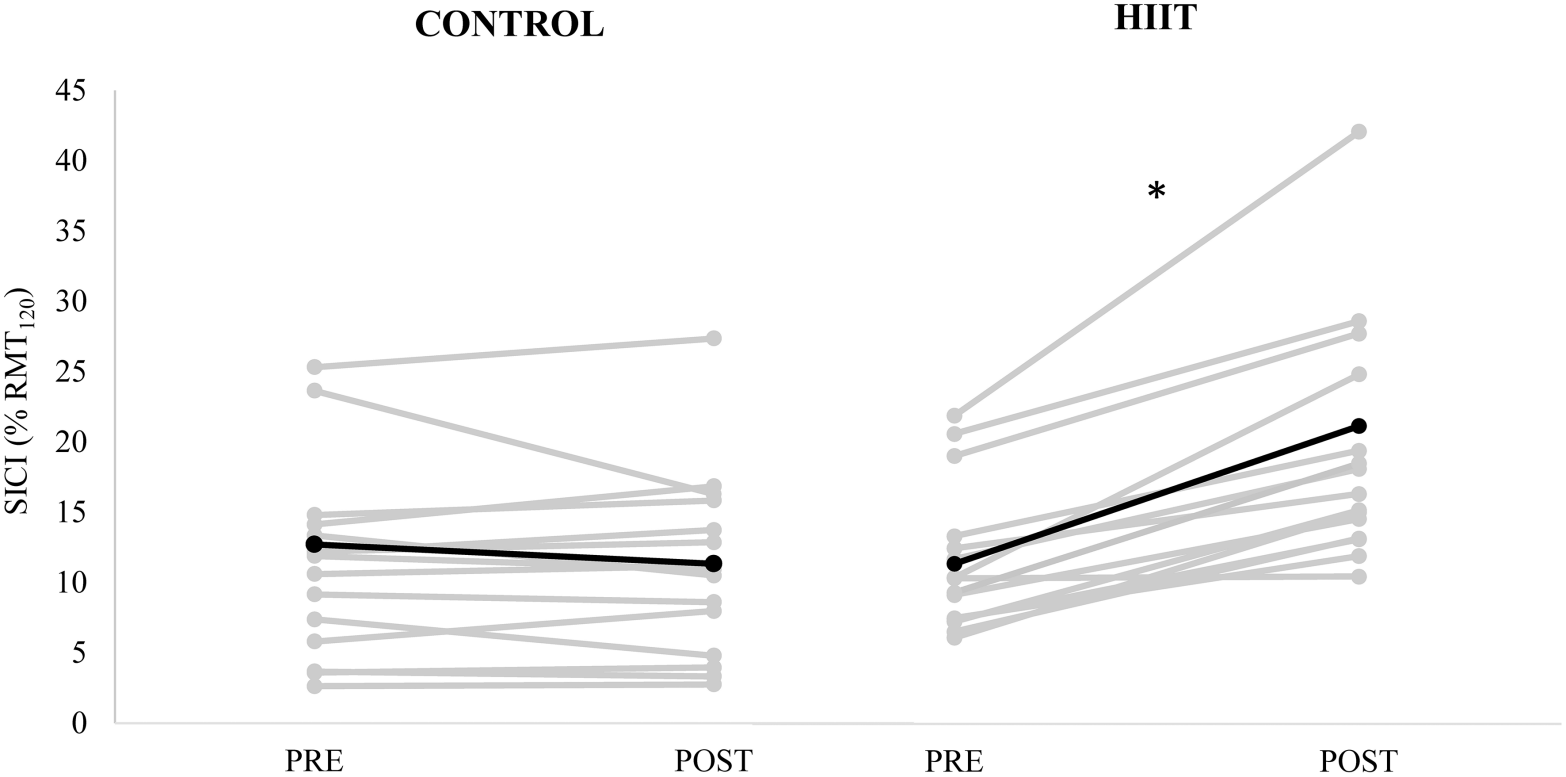
Short-interval intracortical inhibition (conditioned MEP amplitude expressed as % of unconditioned MEP_120_ amplitude) measured PRE and POST for each participant (gray lines) with control condition shown in the left panel, and HIIT condition shown on the right panel. The mean for each condition is depicted by the black line. * denotes a significant condition × time interaction (*p* = 0.002).

Short-interval cortical inhibition, expressed as SICI_(%)_ is shown in Figure 2. There was a significant condition × time interaction [*F*_(2,15)_ = 14.950, *p* = 0.002, *ɳ*_p_^2^ = 0.499]. For the control condition, there was a 10.7% reduction in SICI_(%)_ (from 12.7 ± 8.4 %MEP_120_ to 11.3 ± 6.4 %MEP_120_). For the HIIT condition, there was 86.2% increase in SICI_(%)_ (from 11.4 ± 5.0 %MEP_120_ to 21.1 ± 11.1 %MEP_120_).

### Blood Biomarkers

Table 2 displays the blood biomarkers detected in the immunoassay before and after the intervention for both CONTROL and HIIT conditions. Across all measures, no significant condition × time interactions were detected (all *p* > 0.05). A medium effect size was reported for BDNF (*ɳ*_p_^2^ = 0.219) with a 9.5% reduction following the control condition (from 308.7 ± 97.8 pg/ml to 279.3 ± 102.5 pg/ml) and a 23.6% increase following the HIIT condition (from 362.1 ± 166.1 pg/ml to 447.6 ± 250.2 pg/ml). A medium effect size was also reported for VEGF (*ɳ*_p_^2^ = 0.199) with a 5.0% increase following the control condition (from 127.1 ± 25.4 pg/ml to 133.4 ± 52.0 pg/ml) and a 22.7% increase following the HIIT condition (from 124.2 ± 20.6 pg/ml to 152.4 ± 57.4 pg/ml).

**Table 2:** Blood biomarkers before and after each intervention.

	Control		HIIT				Condition × time interaction		
	Pre	Post	Change	Pre	Post	Change	*F*_(1, 10)_	*p*	*ɳ*_p_^2^
BDNF (pg/ml)	308.7 ± 97.8	279.3 ± 102.5	−9.5%	362.1 ± 166.1	447.6 ± 250.2	23.6%	2.811	0.125	0.219^*^
NGF (pg/L)	387.3 ± 111.6	366.6 ± 91.7	−5.3%	326.4 ± 34.1	356.4 ± 110.5	9.2%	1.285	0.283	0.114
GDNF (pg/ml)	28.1 ± 3.8	27.3 ± 2.9	−2.8%	26.1 ± 1.9	26.5 ± 3.33	1.5%	0.747	0.408	0.069
IGF-1 (ng/ml)	10.6 ± 1.7	10.9 ± 1.5	2.8%	10.3 ± 1.4	9.9 ± 2.2	−3.9%	1.155	0.308	0.104
VEGF (pg/ml)	127.1 ± 25.4	133.4 ± 52.0	5.0%	124.2 ± 20.6	152.4 ± 57.4	22.7%	2.726	0.127	0.199^*^

* *indicates medium effect size (>0.13)*.

## Discussion

The present study investigated how high-intensity exercise alters non-invasive measures of cortical facilitation, inhibition, and blood concentrations of BDNF. Overall, significant interactions were observed for cortical excitability for MEP_120_ (Figure 1A) and intracortical inhibition (Figure 2), where the interactions were driven by an increase in cortical excitability and a decrease in intracortical inhibition for the HIIT condition only. There were no significant differences between conditions for any of the blood biomarkers (Table 2). Although non-significant, HIIT produced a 23.6% increase in BDNF after exercise, whereas control had a −9.5% decrease in blood BDNF (*p* = 0.125, *ɳ*_p_^2^ = 0.219). The medium size effect size combined with the mean changes in BDNF being in the correct direction of our *a-priori* hypothesis, warrants further investigation with a larger sample size.

The significant interaction for MEP_120_, where HIIT increased corticospinal excitability (29.1%) and controls decreased slightly (−6.0%), paired with similar non-significant trends for MEP_140_, suggests that HIIT may prime cortical gray matter and influence GABAergic synaptic transmission into pyramidal neurons. This notion is based on the concept that higher intensity stimulations evoke D-waves which are thought to represent direct excitation of the pyramidal neurons, whereas lower stimulation-intensities are known to evoke I-waves that reflect trans-synaptic activation that originates from cortical level excitatory interneurons, which are controlled by GABAergic interneurons that act into cortico-motoneurons (Ziemann, 2020). Although the stimulation intensity difference between MEP_120_ and MEP_140_ is not great enough to fully differentiate between evoked D-waves from I-waves, the present findings may indicate mechanistic differences. The larger reduction in MEP_120_ may indirectly support the notion that GABAergic inhibition is reduced from these interneurons into cortico-motoneurons in the cerebral cortex. This is supported by the significant reduction in intracortical inhibition, where a significant group × time interaction was observed for SICI_(%)_, driven by an increase in the SICI_(%)_ for the HIIT group (Figure 2). An increase in SICI_(%)_ reflects a reduction in intracortical GABAergic inhibition, which essentially allows pyramidal neurons to become more excitable due to their disinhibited state. Previous literature has established a mechanistic link between reductions in cortical inhibition (i.e., reduction in GABA concentration) and increased long-term potentiation and motor learning (Floyer-Lea et al., 2006; Stagg et al., 2011). With that, HIIT may be an effective method for “priming” the cerebral cortex for enhanced motor learning through decreased inhibitory control of pyramidal neurons.

None of the neurotrophic markers underwent significant changes in the present study. This may be due to the low sample size and the large variability observed across the different neurotrophic factors, as well as the fact that BDNF polymorphisms were not investigated. The *Val66Met* mutation, which occurs in 20–30% of the adult population, can inhibit the release of BDNF following exercise (Lemos et al., 2016), and the presence of this mutation, our sample of participants is unknown. Despite this, non-significant increases of BDNF (23.6%) and VEGF (22.7%) were observed with medium effect sizes (*ɳ*_p_^2^ = 0.219 and 0.199, respectively). Of particular, interest is the 23.6% increase in BDNF for the HIIT group. As an increase in BDNF is known to reduce cortical inhibition and promote excitatory neuroplasticity, the culmination of the findings generally supports this notion. In HIIT, a non-significant increase in BDNF was observed, accompanied by a significant increase in SICI and MEP_120_, all of which are thought to reflect a net reduction in GABAergic synaptic transmission and increased glutamatergic synaptic facilitation resulting in increased corticospinal excitability. The non-significant 22.7% increase in VEGF that was seen in the HIIT group may reflect a modest increase in blood vasculature that facilitates neurovascular coupling in cerebral gray matter, resulting in increased metabolic processes for neuronal firing.

There are several factors to consider that may limit these findings and the generalizability of the work to various populations. First, the effect of exercise intensity on BDNF, IGF-1, and other circulating factors in the blood may also be influenced by the baseline fitness level of the individual (Knaepen et al., 2010; Basso and Suzuki, 2017). Highly active participants exhibit a greater post-exercise increased in these factors when compared to those who have a sedentary lifestyle (Castellano and White, 2008; Zoladz et al., 2008). In this study, we recruited participants who did not meet the Australian physical activity guidelines, which may have contributed to the lack of significant changes in circulating blood factors that were observed. Further, acute aerobic exercise is known to increase circulating levels of BDNF in males to a greater extent than females (Dinoff et al., 2017). With the present study, blood samples were obtained from five male and six female participants. It is possible that the lack of a significant interaction was clouded by the sex differences. In addition, the timing of the post-exercise blood sample (approx. 20 min post-exercise) may have failed to capture the optimal period of the participants response. Few studies report the specific timing of venous sampling, and most studies appear to take single blood samples immediately post-exercise (Knaepen et al., 2010). Some evidence suggests that BDNF levels may return to baseline within 15–20 min of exercise cessation (Vega et al., 2006; Marquez et al., 2015), thus the timing of the post-exercise blood sample in this study (20 min) may have been a limiting factor in the interpretation of our results. Therefore, future studies should consider obtaining blood samples immediately after exercise and also aim to determine optimal exercise dosing for enhancing BDNF in both sexes.

Several TMS studies have previously reported exercise-induced changes in M1 facilitation and inhibition following continuous aerobic training (Singh et al., 2014; Smith et al., 2014). It is not known how exercise type, intensity, and duration might influence the longevity of intracortical modulation. While there have been no specific attempts to assess the time-course duration of exercise-induced changes in intracortical inhibition or facilitation of the non-exercised muscle, Smith et al. (2014) reported reductions in SICI at 15 min post-exercise, but not 30 min post-exercise. In contrast, Singh et al. (2014) showed a significant reduction in inhibition, and increased facilitation at both 0 and 30 min post-exercise, reporting changes of similar magnitude at both time points. It is possible that the intensity of the exercise used in this study, combined with the inclusion of sedentary participants, may have influenced the acute effects of exercise on M1 facilitation and inhibition. Future studies should seek to determine the effects of these factors on acute M1 responses.

In conclusion, the present study investigated the impact of HIIT training on cortical measures of excitability and inhibition, in addition to blood markers of neurotropic factors. An acute increase in MEP_120_, and reduction in SICI for the HIIT group, paired with non-significant medium effect sizes for BDNF and VEGF with mean changes in the direction of our *a-priori* hypotheses, provide indirect evidence that interneuronal synaptic transmission into the pyramidal neurons may be facilitated. These findings support the notion that HIIT provides a potent stimulus for inducing cortical neuroplasticity that may support enhanced motor outcomes with subsequent motor practice. Future research should aim to bridge-the-gap between the present findings with respect to HIIT and its impact on motor learning.

## Data Availability Statement

The raw data supporting the conclusions of this article will be made available by the authors, without undue reservation.

## Ethics Statement

The studies involving human participants were reviewed and approved by Deakin University Human Research Ethics Committee (DUHREC). The patients/participants provided their written informed consent to participate in this study.

## Author Contributions

AH developed the concept, study design, data collection, and analysis. JA, PG, and W-PT contributed to data interpretation and preparation of manuscript. All authors contributed to the article and approved the submitted version.

## Conflict of Interest

The authors declare that the research was conducted in the absence of any commercial or financial relationships that could be construed as a potential conflict of interest.

## Publisher’s Note

All claims expressed in this article are solely those of the authors and do not necessarily represent those of their affiliated organizations, or those of the publisher, the editors and the reviewers. Any product that may be evaluated in this article, or claim that may be made by its manufacturer, is not guaranteed or endorsed by the publisher.

## References

[ref1] Bakeman R. (2005). Recommended effect size statistics for repeated measures designs. Behav. Res. Methods 37, 379–384. doi: 10.3758/BF03192707, PMID: 16405133

[ref2] Basso J. C. Suzuki W. A. (2017). The effects of acute exercise on mood, cognition, neurophysiology, and neurochemical pathways: a review. Brain Plast. 2, 127–152. doi: 10.3233/BPL-160040, PMID: 29765853 PMC5928534

[ref3] Borg G. (1998). Borg's Perceived Exertion and Pain Scales. Champaign, IL: Human Kinetics.

[ref4] Castellano V. White L. J. (2008). Serum brain-derived neurotrophic factor response to aerobic exercise in multiple sclerosis. J. Neurol. Sci. 269, 85–91. doi: 10.1016/j.jns.2007.12.030, PMID: 18275972

[ref5] Church D. D. Hoffman J. R. Mangine G. T. Jajtner A. R. Townsend J. R. Beyer K. S.. (2016). Comparison of high-intensity vs. high-volume resistance training on the BDNF response to exercise. J. Appl. Physiol. 121, 123–128. doi: 10.1152/japplphysiol.00233.2016, PMID: 27231312

[ref6] Cirillo J. Lavender A. P. Ridding M. C. Semmler J. G. (2009). Motor cortex plasticity induced by paired associative stimulation is enhanced in physically active individuals. J. Physiol. 587, 5831–5842. doi: 10.1113/jphysiol.2009.181834, PMID: 19858227 PMC2808543

[ref7] Cortés D. Carballo-Molina O. A. Castellanos-Montiel M. J. Velasco I. (2017). The non-survival effects of glial cell line-derived neurotrophic factor on neural cells. Front. Mol. Neurosci. 10:258. doi: 10.3389/fnmol.2017.00258, PMID: 28878618 PMC5572274

[ref8] Dinoff A. Herrmann N. Swardfager W. Lanctôt K. L. (2017). The effect of acute exercise on blood concentrations of brain-derived neurotrophic factor in healthy adults: a meta-analysis. Eur. J. Neurosci. 46, 1635–1646. doi: 10.1111/ejn.13603, PMID: 28493624

[ref9] El-Sayes J. Turco C. V. Skelly L. E. Locke M. B. Gibala M. J. Nelson A. J. (2020). Acute high-intensity and moderate-intensity interval exercise do not change corticospinal excitability in low fit, young adults. PLoS One 15:e0227581. doi: 10.1371/journal.pone.0227581, PMID: 31978065 PMC6980578

[ref10] Floyer-Lea A. Wylezinska M. Kincses T. Matthews P. M. (2006). Rapid modulation of GABA concentration in human sensorimotor cortex during motor learning. J. Neurophysiol. 95, 1639–1644. doi: 10.1152/jn.00346.2005, PMID: 16221751

[ref11] Gottmann K. Mittmann T. Lessmann V. (2009). BDNF signaling in the formation, maturation and plasticity of glutamatergic and GABAergic synapses. Exp. Brain Res. 199, 203–234. doi: 10.1007/s00221-009-1994-z, PMID: 19777221

[ref12] Hall J. M. Gomez-Pinilla F. Savage L. M. (2018). Nerve growth factor is responsible for exercise-induced recovery of septohippocampal cholinergic structure and function. Front. Neurosci. 12:773. doi: 10.3389/fnins.2018.00773, PMID: 30443202 PMC6222249

[ref13] Inoue D. S. Monteiro P. A. Gerosa-Neto J. Santana P. R. Peres F. P. Edwards K. M.. (2020). Acute increases in brain-derived neurotrophic factor following high or moderate-intensity exercise is accompanied with better cognition performance in obese adults. Sci. Rep. 10:13493. doi: 10.1038/s41598-020-70326-1, PMID: 32778721 PMC7417991

[ref14] Kida H. Mitsushima D. (2018). Mechanisms of motor learning mediated by synaptic plasticity in rat primary motor cortex. Neurosci. Res. 128, 14–18. doi: 10.1016/j.neures.2017.09.008, PMID: 28951322

[ref15] Knaepen K. Goekint M. Heyman E. M. Meeusen R. (2010). Neuroplasticity—exercise-induced response of peripheral brain-derived neurotrophic factor: a systematic review of experimental studies in human subjects. Sports Med. 40, 765–801. doi: 10.2165/11534530-000000000-00000, PMID: 20726622

[ref16] Kou Z. W. Mo J. L. Wu K. W. Qiu M. H. Huang Y. L. Tao F.. (2019). Vascular endothelial growth factor increases the function of calcium-impermeable AMPA receptor GluA2 subunit in astrocytes via activation of protein kinase C signaling pathway. Glia 67, 1344–1358. doi: 10.1002/glia.23609, PMID: 30883902 PMC6594043

[ref17] Lemos J. R. Alves C. R. de Souza S. B. C. Marsiglia J. D. C. Silva M. S. M. Pereira A. C.. (2016). Peripheral vascular reactivity and serum *BDNF* responses to aerobic training are impaired by the *BDNF* Val66Met polymorphism. Physiol. Genomics 48, 116–123. doi: 10.1152/physiolgenomics.00086.2015, PMID: 26603150

[ref18] Lu B. Nagappan G. Lu Y. (2014). BDNF and synaptic plasticity, cognitive function, and dysfunction. Handb. Exp. Pharmacol. 220, 223–250. doi: 10.1007/978-3-642-45106-5_9, PMID: 24668475

[ref19] Lulic T. El-Sayes J. Fassett H. J. Nelson A. J. (2017). Physical activity levels determine exercise-induced changes in brain excitability. PLoS One 12:e0173672. doi: 10.1371/journal.pone.0173672, PMID: 28278300 PMC5344515

[ref20] Mang C. S. Snow N. J. Campbell K. L. Ross C. J. D. Boyd L. A. (2014). A single bout of high-intensity aerobic exercise facilitates response to paired associative stimulation and promotes sequence-specific implicit motor learning. J. Appl. Physiol. 117, 1325–1336. doi: 10.1152/japplphysiol.00498.2014, PMID: 25257866 PMC4254838

[ref21] Marinus N. Hansen D. Feys P. Meesen R. Timmermans A. Spildooren J. (2019). The impact of different types of exercise training on peripheral blood brain-derived neurotrophic factor concentrations in older adults: a meta-analysis. Sports Med. 49, 1529–1546. doi: 10.1007/s40279-019-01148-z, PMID: 31270754

[ref22] Marquez C. M. S. Vanaudenaerde B. Troosters T. Wenderoth N. (2015). High-intensity interval training evokes larger serum BDNF levels compared with intense continuous exercise. J. Appl. Physiol. 119, 1363–1373. doi: 10.1152/japplphysiol.00126.2015, PMID: 26472862

[ref23] Miranda M. Morici J. F. Zanoni M. B. Bekinschtein P. (2019). Brain-derived neurotrophic factor: a key molecule for memory in the healthy and the pathological brain. Front. Cell. Neurosci. 13:363. doi: 10.3389/fncel.2019.00363, PMID: 31440144 PMC6692714

[ref24] Müller S. T. Buchmann A. Haynes M. Ghisleni C. Ritter C. Tuura R.. (2020). Negative association between left prefrontal GABA concentration and BDNF serum concentration in young adults. Heliyon 6:e04025. doi: 10.1016/j.heliyon.2020.e04025, PMID: 32490241 PMC7260440

[ref25] Nofuji Y. Suwa M. Sasaki H. Ichimiya A. Nishichi R. Kumagai S. (2012). Different circulating brain-derived neurotrophic factor responses to acute exercise between physically active and sedentary subjects. J. Sports Sci. Med. 11, 83–88. PMID: 24137066 PMC3737858

[ref26] Opie G. M. Semmler J. G. (2019). Acute exercise at different intensities influences corticomotor excitability and performance of a ballistic thumb training task. Neuroscience 412, 29–39. doi: 10.1016/j.neuroscience.2019.05.049, PMID: 31170481

[ref27] Park H. Poo M.-M. (2013). Neurotrophin regulation of neural circuit development and function. Nat. Rev. Neurosci. 14, 7–23. doi: 10.1038/nrn3379, PMID: 23254191

[ref28] Roig M. Skriver K. Lundbye-Jensen J. Kiens B. Nielsen J. B. (2012). A single bout of exercise improves motor memory. PLoS One 7:e44594. doi: 10.1371/journal.pone.0044594, PMID: 22973462 PMC3433433

[ref29] Rossini P. M. Burke D. Chen R. Cohen L. G. Daskalakis Z. Di Iorio R.. (2015). Non-invasive electrical and magnetic stimulation of the brain, spinal cord, roots and peripheral nerves: basic principles and procedures for routine clinical and research application. An updated report from an I.F.C.N committee. Clin. Neurophysiol. 126, 1071–1107. doi: 10.1016/j.clinph.2015.02.001, PMID: 25797650 PMC6350257

[ref30] Ruegsegger G. N. Booth F. W. (2018). Health benefits of exercise. Cold Spring Harb. Perspect. Med. 8:a029694. doi: 10.1101/cshperspect.a029694, PMID: 28507196 PMC6027933

[ref31] Singh A. M. Duncan R. E. Neva J. L. Staines W. R. (2014). Aerobic exercise modulates intracortical inhibition and facilitation in a nonexercised upper limb muscle. BMC Sports Sci. Med. Rehabil. 6:23. doi: 10.1186/2052-1847-6-23, PMID: 25031838 PMC4100033

[ref32] Smith A. E. Goldsworthy M. R. Garside T. Wood F. M. Ridding M. C. (2014). The influence of a single bout of aerobic exercise on short-interval intracortical excitability. Exp. Brain Res. 232, 1875–1882. doi: 10.1007/s00221-014-3879-z, PMID: 24570388

[ref33] Stagg C. J. Bachtiar V. Johansen-Berg H. (2011). The role of GABA in human motor learning. Curr. Biol. 21, 480–484. doi: 10.1016/j.cub.2011.01.069, PMID: 21376596 PMC3063350

[ref34] Statton M. A. Encarnacion M. Celnik P. Bastian A. J. (2015). A single bout of moderate aerobic exercise improves motor skill acquisition. PLoS One 10:e0141393. doi: 10.1371/journal.pone.0141393, PMID: 26506413 PMC4624775

[ref35] Stavrinos E. L. Coxon J. P. (2017). High-intensity interval exercise promotes motor cortex disinhibition and early motor skill consolidation. J. Cogn. Neurosci. 29, 593–604. doi: 10.1162/jocn_a_01078, PMID: 27897671

[ref36] Vega S. R. Strüder H. K. Wahrmann B. V. Schmidt A. Bloch W. Hollmann W. (2006). Acute BDNF and cortisol response to low intensity exercise and following ramp incremental exercise to exhaustion in humans. Brain Res. 1121, 59–65. doi: 10.1016/j.brainres.2006.08.105, PMID: 17010953

[ref37] Wrigley S. Arafa D. Tropea D. (2017). Insulin-like growth factor 1: at the crossroads of brain development and aging. Front. Cell. Neurosci. 11:14. doi: 10.3389/fncel.2017.00014, PMID: 28203146 PMC5285390

[ref38] Yang T. Nie Z. Shu H. Kuang Y. Chen X. Cheng J.. (2020). The role of BDNF on neural plasticity in depression. Front. Cell. Neurosci. 14:82. doi: 10.3389/fncel.2020.00082, PMID: 32351365 PMC7174655

[ref39] Ziemann U. (2020). I-waves in motor cortex revisited. Exp. Brain Res. 238, 1601–1610. doi: 10.1007/s00221-020-05764-4, PMID: 32185405 PMC7413903

[ref40] Zoladz J. A. Pilc A. Majerczak J. Grandys M. Zapart-Bukowska J. Duda K. (2008). Endurance training increases plasma brain-derived neurotrophic factor concentration in young healthy men. J. Physiol. Pharmacol. 59, 119–132. PMID: 19258661

